# ChatGPT and Psychiatric Discharge Summaries: The Assistant We’ve Been Dreaming of?

**DOI:** 10.1192/j.eurpsy.2025.519

**Published:** 2025-08-26

**Authors:** J. M. De Castro, M. Sant’Ovaia, C. Martins, A. C. Ramos, M. Palma

**Affiliations:** 1 Serviço de Psiquiatria, Unidade Local de Saúde de Amadora/Sintra, Amadora, Portugal

## Abstract

**Introduction:**

ChatGPT is a language model based on artificial intelligence (AI) that is designed to generate human-like text. It offers potential applications in automating and simplification of clinical documentation tasks, addressing the increasing administrative burden that contributes to high rates of burnout in psychiatry. As discharge summaries are typically structured and repetitive, the use of ChatGPT to automate this task could offer significant benefits, such as reducing clinical workload, improving summary quality, and preventing delays in patient discharges. However, concerns about reliability, accuracy, and ethical considerations persist.

**Objectives:**

Explore the feasibility and implications of using ChatGPT to assist in writing discharge summaries in psychiatric settings.

**Methods:**

A narrative review was conducted by searching PubMed and Google Scholar with the keywords “ChatGPT”, “discharge” and “psychiatry”. Relevant articles, including empirical studies, case reports, reviews, and expert opinions were selected.

**Results:**

We found only one empirical study that evaluated psychiatric discharge summaries generated with ChatGPT-4: human-written discharge summaries were rated significantly higher in quality than those generated by ChatGPT; the ChatGPT summaries fell short, particularly in coherence and specificity of formulations, though they performed reasonably well in summarizing relevant case information. Most of the literature consisted of theoretical discussions and expert opinions related to the broader use of AI in psychiatry. Despite this, the potential benefits, such as improving the efficiency and consistency of documentation, were frequently highlighted. However, concerns related to accuracy, the need for clinician oversight, and ethical implications were consistently noted.

**Conclusions:**

ChatGPT shows promise in assisting with the generation of psychiatric discharge summaries, potentially alleviating the documentation burden faced by clinicians. However, further refinement of the model, integration with electronic health records, and the establishment of clear ethical safeguards are necessary for its safe and effective use. The current lack of empirical evidence highlights the need for targeted research that should also address challenges related to data governance, patient acceptance, and error management. Additionally, studies should evaluate the direct impact on clinician workload and compare the quality of AI-generated summaries with those written by psychiatrists and residents. Such research will be essential to facilitate the broader integration of ChatGPT in real-world psychiatric practice.

**Disclosure of Interest:**

None Declared

